# Ethyl 3-amino-5-bromo-1-benzo­furan-2-carboxyl­ate

**DOI:** 10.1107/S1600536813010209

**Published:** 2013-04-20

**Authors:** A. J. Yamuna, P. Karunakar, C. R. Girija, V. P. Vaidya, V. Krishnamurthy

**Affiliations:** a Department of Chemistry, Kuvempu University, Jnana Sahyadri, Shankaraghatta 577 451, India; b Department of Biotechnology, PES Institute of Technology, BSK III Stg, Bangalore 560 085, India; c Department of Chemistry, SSMRV College, 4th T Block, Jayanagar, Bangalore 560 041, India

## Abstract

The title compound, C_11_H_10_BrNO_3_, is close to planar with the benzo­furan unit and the ester group subtending a dihedral angle of 5.25 (2)°. The mol­ecular structure features an intra­molecular N—H⋯O inter­action. In the crystal, N—H⋯O hydrogen bonds involving carboxyl O-atom acceptors generate a chain extending along [201].

## Related literature

For the biological activity of benzo­furan derivatives, see: Oter *et al.* (2007 ▶); Habermann *et al.* (1999 ▶). For a similar structure, see: Karunakar *et al.* (2013 ▶).
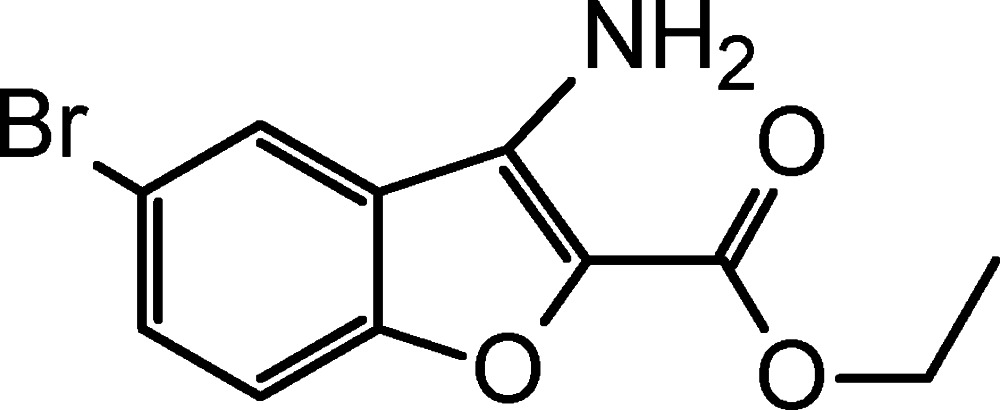


## Experimental

### 

#### Crystal data

C_11_H_10_BrNO_3_
*M* *_r_* = 284.11
Monoclinic, 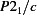

*a* = 5.775 (5) Å
*b* = 25.550 (2) Å
*c* = 7.640 (1) Å
β = 98.292 (1)°
*V* = 1115.5 (10) Å^3^
*Z* = 4
Mo *K*α radiation
μ = 3.68 mm^−1^
*T* = 293 K
0.20 × 0.15 × 0.10 mm

#### Data collection

Bruker Kappa APEXII CCD diffractometer
Absorption correction: multi-scan (*SADABS*; Bruker, 2008 ▶) *T*_min_ = 0.591, *T*_max_ = 0.732
10542 measured reflections
1957 independent reflections
1658 reflections with *I* > 2σ(*I*)
*R*_int_ = 0.025

#### Refinement

*R*[*F* ^2^ > 2σ(*F* ^2^)] = 0.033
*wR*(*F* ^2^) = 0.076
*S* = 1.08
1957 reflections
154 parameters
1 restraint
H atoms treated by a mixture of independent and constrained refinement
Δρ_max_ = 0.42 e Å^−3^
Δρ_min_ = −0.34 e Å^−3^

### 

Data collection: *APEX2* (Bruker, 2004 ▶); cell refinement: *SAINT-Plus* (Bruker, 2004 ▶); data reduction: *SAINT-Plus* and *XPREP* (Bruker, 2004 ▶); program(s) used to solve structure: *SIR92* (Altomare *et al.*, 1993 ▶); program(s) used to refine structure: *SHELXL97* (Sheldrick, 2008 ▶); molecular graphics: *ORTEP-3 for Windows* (Farrugia, 2012 ▶) and *Mercury* (Macrae *et al.*, 2006 ▶); software used to prepare material for publication: *SHELXL97*.

## Supplementary Material

Crystal structure: contains datablock(s) I, global. DOI: 10.1107/S1600536813010209/zs2255sup1.cif

Structure factors: contains datablock(s) I. DOI: 10.1107/S1600536813010209/zs2255Isup2.hkl

Supplementary material file. DOI: 10.1107/S1600536813010209/zs2255Isup3.cdx

Supplementary material file. DOI: 10.1107/S1600536813010209/zs2255Isup4.cml

Additional supplementary materials:  crystallographic information; 3D view; checkCIF report

## Figures and Tables

**Table 1:** Hydrogen-bond geometry (Å, °)

*D*—H⋯*A*	*D*—H	H⋯*A*	*D*⋯*A*	*D*—H⋯*A*
N1—H1*A*⋯O2^i^	0.82 (2)	2.17 (2)	2.977 (4)	171 (3)
N1—H1*B*⋯O3	0.82 (2)	2.31 (3)	2.835 (4)	123 (3)

Symmetry code: (i) 
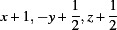
.

**Fig. 1.**
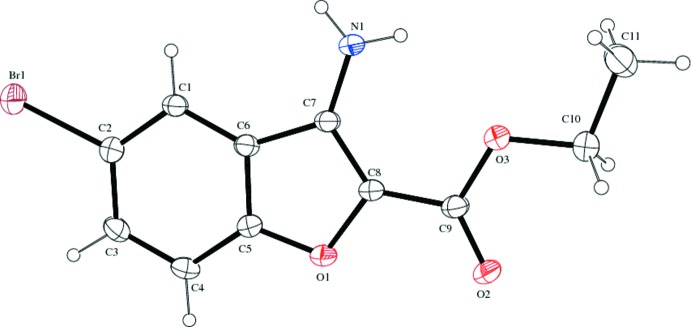
Molecular conformation and atom numbering scheme for the title compound, with displacement ellipsoids drawn at the 50% probability level.

**Fig. 2.**
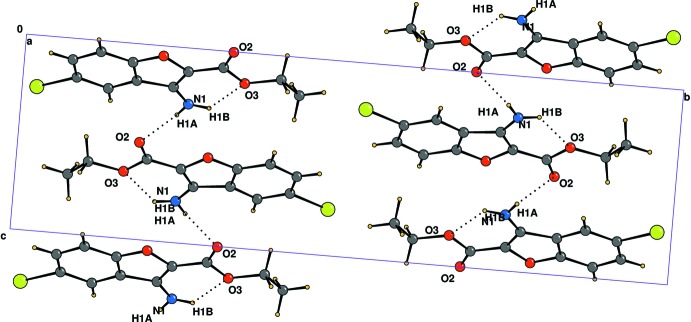
The crystal packing in the unit cell viewed down a. Hydrogen bonds are drawn as dashed lines. For symmetry code (i), see Table 1.

## supplementary crystallographic information

### Comment

Substituted benzofurans find their applications in different fields such as fluorescent sensors (Oter *et al.*, 2007), antioxidants, brightening agents and as drugs and agricultural chemicals (Habermann *et al.*, 1999). In the title compound, C_11_H_10_BrNO_3_, the ester group is close to coplanar with the benzofuran plane [dihedral angle = 5.25 (2)°] which compares with 7.84 (2)° in the previously reported analogous compound ethyl 5-bromo-3-ethoxycarbonylamino-1-benzofuran-2-carboxylate (Karunakar *et al.*, 2013). The structure is stabilized by an intramolecular amine N1—H1*B*···O3 interaction (Table 1) while an intermolecular N1—H*A*···O2^i^ hydrogen-bond to a carboxyl O-atom acceptor generates a one-dimensional chain structure extending along [201] (Fig. 2).

### Experimental

The mixture of 5-bromo-2-hydroxybenzonitrile (1.98 g, 0.01 mol), ethyl chloroacetate (1 ml, 0.01 mol) and potassium carbonate (2.76 g, 0.02 mol) in 5 ml of DMF was refluxed for 90 min. The potassium carbonate was removed by filtration and crushed ice was added to the filtrate, with constant stirring. The solid separated was filtered, dried and recrystallized from ethanol. Yeild: 87% (0.247 g); m.p. 154–156 °C.

### Refinement

All carbon-bound hydrogen atoms were placed in calculated positions with C—H distances of 0.93 - 0.97 Å and refined as riding with *U*_iso_(H) =x*U*_eq_(C), where *x* = 1.5 for methyl H-atoms and *x* = 1.2 for all other H-atoms. The N-bound H atom positions were determined from a difference electron density map and refined freely.

### Crystal data

**Table:**

C_11_H_10_BrNO_3_	*F*(000) = 568
*M**_r_* = 284.11	*D*_x_ = 1.692 Mg m^−^^3^
Monoclinic, *P*2_1_/*c*	Melting point = 427–429 K
Hall symbol: -P 2ybc	Mo *K*α radiation, λ = 0.71073 Å
*a* = 5.775 (5) Å	Cell parameters from 4034 reflections
*b* = 25.550 (2) Å	θ = 3.1–25.0°
*c* = 7.640 (1) Å	µ = 3.68 mm^−^^1^
β = 98.292 (1)°	*T* = 293 K
*V* = 1115.5 (10) Å^3^	Block, yellow
*Z* = 4	0.20 × 0.15 × 0.10 mm

### Data collection

**Table:**

Bruker Kappa APEXII CCD diffractometer	1957 independent reflections
Radiation source: fine-focus sealed tube	1658 reflections with *I* > 2σ(*I*)
Graphite monochromator	*R*_int_ = 0.025
ω and φ scans	θ_max_ = 25.0°, θ_min_ = 2.8°
Absorption correction: multi-scan (*SADABS*; Bruker, 2008)	*h* = −6→6
*T*_min_ = 0.591, *T*_max_ = 0.732	*k* = −30→30
10542 measured reflections	*l* = −9→9

### Refinement

**Table:**

Refinement on *F*^2^	Primary atom site location: structure-invariant direct methods
Least-squares matrix: full	Secondary atom site location: difference Fourier map
*R*[*F*^2^ > 2σ(*F*^2^)] = 0.033	Hydrogen site location: inferred from neighbouring sites
*wR*(*F*^2^) = 0.076	H atoms treated by a mixture of independent and constrained refinement
*S* = 1.08	*w* = 1/[σ^2^(*F*_o_^2^) + (0.0258*P*)^2^ + 1.2998*P*] where *P* = (*F*_o_^2^ + 2*F*_c_^2^)/3
1957 reflections	(Δ/σ)_max_ < 0.001
154 parameters	Δρ_max_ = 0.42 e Å^−^^3^
1 restraint	Δρ_min_ = −0.34 e Å^−^^3^

### Special details

**Table:**

Geometry. All e.s.d.'s (except the e.s.d. in the dihedral angle between two l.s. planes) are estimated using the full covariance matrix. The cell e.s.d.'s are taken into account individually in the estimation of e.s.d.'s in distances, angles and torsion angles; correlations between e.s.d.'s in cell parameters are only used when they are defined by crystal symmetry. An approximate (isotropic) treatment of cell e.s.d.'s is used for estimating e.s.d.'s involving l.s. planes.
Refinement. Refinement of *F*^2^ against ALL reflections. The weighted *R*-factor *wR* and goodness of fit *S* are based on *F*^2^, conventional *R*-factors *R* are based on *F*, with *F* set to zero for negative *F*^2^. The threshold expression of *F*^2^ > σ(*F*^2^) is used only for calculating *R*-factors(gt) *etc*. and is not relevant to the choice of reflections for refinement. *R*-factors based on *F*^2^ are statistically about twice as large as those based on *F*, and *R*- factors based on ALL data will be even larger.

### Fractional atomic coordinates and isotropic or equivalent isotropic displacement parameters (Å^2^)

**Table:**

	*x*	*y*	*z*	*U*_iso_*/*U*_eq_	
C1	0.7527 (5)	0.13047 (11)	0.2409 (4)	0.0367 (6)	
H1	0.9027	0.1351	0.3018	0.044*	
C2	0.6640 (5)	0.08164 (12)	0.1981 (4)	0.0400 (7)	
C3	0.4395 (5)	0.07344 (12)	0.1094 (4)	0.0449 (8)	
H3	0.3859	0.0395	0.0855	0.054*	
C4	0.2971 (5)	0.11501 (13)	0.0572 (4)	0.0457 (8)	
H4	0.1464	0.1102	−0.0023	0.055*	
C5	0.3869 (5)	0.16441 (12)	0.0968 (4)	0.0373 (7)	
C6	0.6077 (5)	0.17270 (11)	0.1889 (4)	0.0339 (6)	
C7	0.6341 (5)	0.22874 (11)	0.2061 (4)	0.0345 (6)	
C8	0.4298 (5)	0.24944 (12)	0.1214 (4)	0.0385 (7)	
C9	0.3548 (5)	0.30198 (12)	0.0857 (4)	0.0402 (7)	
C10	0.4688 (6)	0.39058 (12)	0.1228 (4)	0.0464 (8)	
H10A	0.3386	0.4018	0.1810	0.056*	
H10B	0.4292	0.3967	−0.0032	0.056*	
C11	0.6871 (6)	0.41993 (14)	0.1950 (5)	0.0613 (10)	
H11A	0.7188	0.4152	0.3208	0.092*	
H11B	0.6657	0.4565	0.1686	0.092*	
H11C	0.8163	0.4069	0.1416	0.092*	
N1	0.8245 (5)	0.25350 (11)	0.2871 (4)	0.0472 (7)	
O1	0.2739 (3)	0.21006 (8)	0.0521 (3)	0.0446 (5)	
O2	0.1709 (4)	0.31544 (9)	0.0025 (3)	0.0579 (6)	
O3	0.5187 (4)	0.33597 (8)	0.1573 (3)	0.0463 (5)	
Br1	0.85639 (7)	0.022292 (13)	0.26302 (6)	0.06241 (16)	
H1A	0.930 (5)	0.2374 (11)	0.347 (4)	0.045 (9)*	
H1B	0.821 (6)	0.2852 (9)	0.299 (5)	0.058 (11)*	

### Atomic displacement parameters (Å^2^)

**Table:**

	*U*^11^	*U*^22^	*U*^33^	*U*^12^	*U*^13^	*U*^23^
C1	0.0319 (14)	0.0367 (16)	0.0390 (16)	−0.0032 (13)	−0.0029 (12)	0.0002 (13)
C2	0.0411 (17)	0.0360 (16)	0.0422 (18)	0.0023 (13)	0.0036 (13)	0.0003 (13)
C3	0.0427 (17)	0.0379 (17)	0.0527 (19)	−0.0096 (14)	0.0020 (14)	−0.0093 (15)
C4	0.0344 (16)	0.0486 (19)	0.0508 (19)	−0.0091 (14)	−0.0053 (14)	−0.0077 (15)
C5	0.0314 (15)	0.0382 (16)	0.0406 (16)	0.0011 (13)	−0.0005 (12)	−0.0001 (13)
C6	0.0321 (15)	0.0359 (16)	0.0322 (15)	−0.0034 (12)	−0.0002 (12)	0.0002 (12)
C7	0.0295 (15)	0.0373 (16)	0.0347 (16)	−0.0027 (12)	−0.0024 (12)	0.0003 (12)
C8	0.0309 (15)	0.0376 (16)	0.0434 (17)	−0.0012 (13)	−0.0067 (13)	−0.0009 (13)
C9	0.0313 (16)	0.0427 (17)	0.0438 (17)	−0.0016 (13)	−0.0036 (13)	0.0037 (14)
C10	0.0490 (19)	0.0368 (17)	0.0522 (19)	0.0022 (14)	0.0034 (15)	0.0026 (15)
C11	0.061 (2)	0.050 (2)	0.072 (3)	−0.0128 (17)	0.0062 (19)	−0.0075 (18)
N1	0.0345 (14)	0.0365 (16)	0.0635 (19)	−0.0002 (13)	−0.0171 (13)	0.0010 (14)
O1	0.0316 (11)	0.0415 (12)	0.0556 (13)	−0.0025 (9)	−0.0107 (9)	−0.0014 (10)
O2	0.0409 (13)	0.0439 (13)	0.0798 (17)	0.0019 (10)	−0.0221 (12)	0.0092 (12)
O3	0.0402 (12)	0.0369 (12)	0.0568 (13)	0.0004 (9)	−0.0097 (10)	0.0023 (10)
Br1	0.0607 (2)	0.0360 (2)	0.0857 (3)	0.00615 (17)	−0.00581 (18)	−0.00165 (18)

### Geometric parameters (Å, º)

**Table:**

C1—C2	1.370 (4)	C8—O1	1.402 (3)
C1—C6	1.388 (4)	C8—C9	1.425 (4)
C1—H1	0.9300	C9—O2	1.207 (3)
C2—C3	1.389 (4)	C9—O3	1.342 (3)
C2—Br1	1.903 (3)	C10—O3	1.441 (4)
C3—C4	1.367 (4)	C10—C11	1.501 (5)
C3—H3	0.9300	C10—H10A	0.9700
C4—C5	1.381 (4)	C10—H10B	0.9700
C4—H4	0.9300	C11—H11A	0.9600
C5—O1	1.356 (3)	C11—H11B	0.9600
C5—C6	1.381 (4)	C11—H11C	0.9600
C6—C7	1.444 (4)	N1—H1A	0.82 (2)
C7—N1	1.340 (4)	N1—H1B	0.82 (2)
C7—C8	1.368 (4)		
			
C2—C1—C6	116.7 (3)	C7—C8—C9	132.3 (3)
C2—C1—H1	121.6	O1—C8—C9	116.3 (2)
C6—C1—H1	121.6	O2—C9—O3	123.1 (3)
C1—C2—C3	123.0 (3)	O2—C9—C8	126.1 (3)
C1—C2—Br1	118.6 (2)	O3—C9—C8	110.8 (2)
C3—C2—Br1	118.4 (2)	O3—C10—C11	106.3 (3)
C4—C3—C2	120.3 (3)	O3—C10—H10A	110.5
C4—C3—H3	119.8	C11—C10—H10A	110.5
C2—C3—H3	119.8	O3—C10—H10B	110.5
C3—C4—C5	117.1 (3)	C11—C10—H10B	110.5
C3—C4—H4	121.5	H10A—C10—H10B	108.7
C5—C4—H4	121.5	C10—C11—H11A	109.5
O1—C5—C6	111.8 (2)	C10—C11—H11B	109.5
O1—C5—C4	125.4 (3)	H11A—C11—H11B	109.5
C6—C5—C4	122.8 (3)	C10—C11—H11C	109.5
C5—C6—C1	120.1 (3)	H11A—C11—H11C	109.5
C5—C6—C7	106.0 (2)	H11B—C11—H11C	109.5
C1—C6—C7	133.9 (3)	C7—N1—H1A	121 (2)
N1—C7—C8	129.1 (3)	C7—N1—H1B	119 (3)
N1—C7—C6	125.4 (3)	H1A—N1—H1B	118 (3)
C8—C7—C6	105.5 (2)	C5—O1—C8	105.2 (2)
C7—C8—O1	111.4 (3)	C9—O3—C10	116.2 (2)
			
C6—C1—C2—C3	−0.9 (5)	C1—C6—C7—C8	177.9 (3)
C6—C1—C2—Br1	179.1 (2)	N1—C7—C8—O1	179.3 (3)
C1—C2—C3—C4	1.3 (5)	C6—C7—C8—O1	0.4 (3)
Br1—C2—C3—C4	−178.7 (2)	N1—C7—C8—C9	2.5 (6)
C2—C3—C4—C5	0.0 (5)	C6—C7—C8—C9	−176.4 (3)
C3—C4—C5—O1	178.4 (3)	C7—C8—C9—O2	176.9 (3)
C3—C4—C5—C6	−1.7 (5)	O1—C8—C9—O2	0.2 (5)
O1—C5—C6—C1	−177.9 (3)	C7—C8—C9—O3	−2.8 (5)
C4—C5—C6—C1	2.1 (5)	O1—C8—C9—O3	−179.5 (2)
O1—C5—C6—C7	1.1 (3)	C6—C5—O1—C8	−0.8 (3)
C4—C5—C6—C7	−178.9 (3)	C4—C5—O1—C8	179.2 (3)
C2—C1—C6—C5	−0.7 (4)	C7—C8—O1—C5	0.3 (3)
C2—C1—C6—C7	−179.4 (3)	C9—C8—O1—C5	177.6 (3)
C5—C6—C7—N1	−179.8 (3)	O2—C9—O3—C10	−2.8 (4)
C1—C6—C7—N1	−1.1 (5)	C8—C9—O3—C10	176.9 (3)
C5—C6—C7—C8	−0.8 (3)	C11—C10—O3—C9	−172.8 (3)

### Hydrogen-bond geometry (Å, º)

**Table:**

*D*—H···*A*	*D*—H	H···*A*	*D*···*A*	*D*—H···*A*
N1—H1*A*···O2^i^	0.82 (2)	2.17 (2)	2.977 (4)	171 (3)
N1—H1*B*···O3	0.82 (2)	2.31 (3)	2.835 (4)	123 (3)

Symmetry code: (i) *x*+1, −*y*+1/2, *z*+1/2.
